# Association of Iron Supplementation Programs with Iron-Deficiency Anemia Outcomes among Children in Brazil

**DOI:** 10.3390/nu13051524

**Published:** 2021-04-30

**Authors:** Carolina Thalya da Silva Paulino, Marislei Nishijima, Flavia Mori Sarti

**Affiliations:** 1 School of Arts, Sciences and Humanities, University of Sao Paulo, Sao Paulo 03828-000, Brazil; carolinathalyas@usp.br; 2 Institute of International Relations, University of Sao Paulo, Sao Paulo 03828-000, Brazil; marislei@usp.br

**Keywords:** iron-deficiency anemia, child health, national health programs

## Abstract

Anemia remains a condition with high prevalence in populations worldwide, and the prevalence of anemia among children under five years old in Brazil is approximately 40%, being higher in communities marked by social inequities. Diverse government programs during recent decades targeted iron-deficiency anemia, considering its impacts throughout the lifetime. The objective of this study was to investigate the effects of two government iron supplementation programs on health outcomes related to iron-deficiency anemia among children up to 4 years old in Brazilian municipalities. A longitudinal panel encompassing data from 5570 municipalities from 1998 to 2019 was investigated using a difference-in-differences framework with multiple interventions and distinct times of adhesion, and fixed-effects models were estimated to control for invariant municipal characteristics throughout the period in order to ensure comparability. The results indicate significant effects of the federal programs in reducing hospitalizations and lengths of stay due to iron-deficiency anemia, especially in non-poor municipalities. There was complementarity in the effects of the programs; however, neither of the programs influenced mortality rates. Thus, it is important to consider possible improvements in the operationalization of the programs, in order to achieve better results in the reduction of severe iron-deficiency anemia among children up to 4 years old.

## 1. Introduction

Anemia remains a condition with high prevalence in populations worldwide. It is predominantly associated with nutritional status and socioeconomic conditions or hereditary diseases. According to the World Health Organization (WHO), anemia is a result of low hemoglobin content in the blood, due to the lack of one or more essential nutrients [1]. The majority of anemia cases are linked to iron deficiency—named iron-deficiency anemia—resulting from low intake of iron-rich foods in the diet, or from substantial blood loss [2].

It is well established that iron-deficiency anemia is an important limiting factor for child growth and development [3,4,5,6,7], especially among low-income populations in developing countries [2,8,9]. Usually, the peak of iron-deficiency anemia among infants occurs between 1 and 3 years of age, and is significantly associated with substantial morbidity, higher health care utilization, and cognitive deficits in later life [10].

Thus, the higher prevalence of iron-deficiency anemia among pre-school children compromises their short-term health outcomes and long-term achievements throughout life—especially schooling and income—and establishes an intergenerational transmission of poverty due to its effects on human capital development [11,12].

According to recent estimates from systematic reviews and meta-analyses, the pooled prevalence of anemia among children under five years old in Brazil is approximately 40% [13], being higher in children living in communities marked by social inequities (51.6%) throughout the country [14]. Iron-deficiency anemia has been a serious public health problem targeted by diverse government programs during recent decades, focusing on vulnerable populations through primary health care initiatives at the municipal level within the Brazilian Unified Health System (SUS) [15]. Two major programs tackling iron-deficiency anemia in pre-school children nationwide are the National Iron Supplementation Program (PNSF) and the Strategy of Food Fortification with Micronutrients for Children (NutriSUS) [11,15].

The PNSF has been implemented through the distribution of ferrous sulfate acquired by the Brazilian Ministry of Health and distributed to municipalities adhering to the program since 2005, being decentralized to the municipalities in 2013. The ferrous sulfate supplements are distributed through primary health care facilities to children aged 6–24 months, and to women from the beginning of the pregnancy until 3 months after the child’s birth [15].

The NutriSUS is based on the distribution of multi-micronutrient supplements (including iron and zinc) for the fortification of foods at home for 60 days per semester, for children between 6 and 48 months of age; these have been acquired by the federal government since 2014, and distributed to primary health care facilities in the municipalities, which are responsible for their delivery to daycare centers [11].

According to Baltussen et al. [16], strategies based on iron supplementation may have larger impacts on population health than an iron fortification program, although iron fortification of foods usually consumed by the population may be less costly than iron supplementation. However, it is important to highlight that both iron supplementation and fortification present high cost-effectiveness in tackling iron-deficiency anemia worldwide.

On the other hand, it is important to highlight a lack of robust evidence on the prevalence and the health outcomes of iron-deficiency anemia in population-level studies [17], including the long-term effects of national policies addressing iron-deficiency anemia [18,19], particularly in developing countries. Thus, the present study contributes with evidence on the occurrence of iron-deficiency anemia diagnosed at the hospital level, and the associated impacts of federal supplementation programs on health outcomes related to iron-deficiency anemia among children up to 4 years old in Brazilian municipalities from 1998 to 2019 in a developing country, Brazil.

Considering the wide impacts of iron-deficiency anemia throughout the lifetime, the results of this study may support the identification of governmental strategies of iron supplementation suited for developing countries dealing with anemia among children, given the identification of the impact of the cumulative benefits derived from public health programs conducted simultaneously during recent decades.

## 2. Materials and Methods

### 2.1. Data

The data comprised annual information at the municipal level from publicly available sources gathered to study the effects of two programs designed to tackle iron-deficiency anemia among children in Brazil: the PNSF in 2005, and its decentralization in 2013; and the adoption of multi-micronutrient supplements by the NutriSUS in 2014. The panel data include information on 5570 municipalities during the period from 1998 to 2019.

Data at the municipal level included information on the distribution of ferrous sulfate in two administration modalities (pills or syrup) in the PNSF from 2005 to 2016, and data on the population coverage of multi-micronutrient supplements by the NutriSUS from 2014 to 2016, obtained from the Brazilian Ministry of Health through requirement under government transparency legislation (the Law of Access to Information 12,527 of 18 November 2011). Supplementary data on population coverage of the PNSF and NutriSUS from 2017 to 2019 were obtained via the open-data platform of the Brazilian Ministry of Health.

Information on health outcomes during hospital care in municipalities was gathered from the Department of Informatics of the Brazilian Unified Health System (DATASUS), encompassing hospital admissions, days of hospitalization, and deaths due to iron-deficiency anemia among children up to 4 years old. Information on other health conditions potentially affecting the outcomes of iron supplementation programs (such as the occurrence of infectious intestinal diseases) was also included in the dataset.

Additional data were gathered via platforms of the Brazilian government to create control variables for other interventions against anemia (intravenous iron applications) and undernutrition (medical appointments at home)—obtained from the DATASUS—and population count and socioeconomic status (Gross Domestic Product, GDP) in the municipality, obtained from the Brazilian Institute for Geography and Statistics (IBGE).

Intravenous iron applications have been performed in cases of extreme anemia occurring in individuals who present gastrointestinal problems that may interfere with the absorption of iron administered orally during treatment of anemia, and medical appointments at home represent a proxy for coverage of the Family Health Strategy program—a community healthcare program delivered by teams of health professionals serving 100–150 households with monthly visits, regardless of need or demand [20].

Population counts per municipality were included in the dataset in order to account for economies of scale in the distribution of supplements to children, and the GDP of the municipalities was incorporated into the analysis so as to control for local socioeconomic conditions that may influence the decisions of adherence by, and the health outcomes of, the PNSF and NutriSUS.

### 2.2. Variables

The outcome variables in the study included hospitalization rate, mean length of stay per hospitalization, and mortality rate due to iron-deficiency anemia among children up to 4 years old. Data on hospitalizations and deaths due to iron-deficiency anemia among children up to 4 years old were converted into hospitalization and mortality rates per 1000 individuals using data on the population up to 4 years old of the municipality. Information on days of hospitalization due to iron-deficiency anemia was converted into the mean length of stay per hospitalization, by dividing the total days of hospitalization registered among children up to 4 years old by the number of hospitalizations.

The target variables of interest in the study referred to the coverage of programs directed towards iron-deficiency anemia among children up to 4 years old in Brazil. The distribution of two administration modalities (pills or syrup) of ferrous sulfate by the PNSF from the federal government to the Brazilian municipalities was converted into PNSF coverage by dividing the supplements distributed per member of the municipal population up to 4 years old. Subsequently, the PNSF and NutriSUS coverage were converted into the municipalities’ relative coverage rates per year using feature scaling, i.e., a rescaling process using the minimum and maximum values of the annual distribution of supplements from the Brazilian Ministry of Health to the municipalities, in order to ensure comparability between diverse units of measurement of the supplements.

Control variables for other interventions against anemia (intravenous iron applications), undernutrition (medical appointments at home, a proxy for the Family Health Strategy program), and other health conditions potentially influencing the results of iron supplementation (e.g., the occurrence of infectious intestinal diseases) were converted into procedures, appointments, and incidence per 1000 inhabitants. Information on the socioeconomic status of the population (GDP) was converted into a logarithm of GDP per capita.

Additional control variables in the analysis included: binary variables referring to the joint effects of PNSF decentralization with the implementation of the NutriSUS (from 2014 onwards); a logarithm of the population of the municipality; and year and state binary variables (including crossed variables for year and state) to control for state-level programs (Table 1).

### 2.3. Models

Considering that the programs created to tackle iron-deficiency anemia among children have been implemented in different time periods, with staggered adhesion by municipalities and superposition of interventions, we adopted a difference-in-differences (DD) framework, with multiple interventions at distinct times of adhesion in its version of intensity [21,22]. In the last case, instead of using 1 to indicate the treated group, we adopted a value between 0 and 1 to indicate the intensity of the coverage of the program of interest. The DD framework allowed us to control the differences before and after an intervention, and between treated and non-treated (a control group) municipalities, eliminating effects not connected to the intervention, as well as effects caused by different levels of a variable before the intervention.

Equation (1) illustrates the DD structure, where the variable *PNSF_it_* synthesizes the programs’ staggered adhesion by the municipalities over time and the degree of coverage after the adhesion. We also included two other interventions in the model: the NutriSUS program beginning in 2014; and the decentralization of the PNSF program that was implemented in the same year. In the latter case, we includeed the *NUSUS_it_* variable, which is the program coverage to account for the effect of the program; the *POS*_2014_, which is a binary variable that accounts for both the time of NutriSUS and that of PNSF decentralization, being 0 before 2014 and 1 after; and the crossed term *POS*_2014_*. PNSF_it_*, which measures the effect of the PNSF’s decentralization. These variables, along with the set of year dummies (included in the A matrix of variables), provide the DD framework of multiple interventions and multiple time intervention. Since the design and planning of the programs were conducted under the decisions of the Brazilian federal government, we take advantage of this, considering the programs as external shocks for municipalities. This allows us to identify the effects of the programs on the health indicators. The estimates are performed in ordinary least squares:

*y_it_* = *β*_0_ + *β*_1_*PNSF_it_* + *β*_2_*NSUS_it_* + *β*_3_*POS*_2014_ + *β*_4_*POS*_2014_. *PNSF_it_* + *A*’*B* + *µ_it_* (1)

Thus, in Equation (1), the coefficients of interest are *β*_1,_ which measures the effect of the PNSF program on child nutrition in the year *t* and municipality *i*; *β*_2,_ which measures the effect of the NutriSUS program; and *β*_4_, which accounts for the effect of the PNSF’s decentralization to the municipalities from 2014 onwards. The dependent variables (*y_it_*) represent the hospitalization rate due to iron-deficiency anemia per 1000 children up to 4 years old, the mean length of stay per hospitalization due to anemia among children up to 4 years old, and the mortality rate due to anemia per 1000 children up to 4 years old. Matrix *A* includes all control variables (see Table 1), while vector *B* includes the associated coefficients. Finally, *µ_it_* represents the orthogonal error in the model.

However, even considering the programs and policies as exogenous shocks, given the potential endogeneity of adhesion and timing of adhesion, fixed effect (FE) models were also estimated in order to control for invariant municipal characteristics throughout the period, making municipalities more similar for comparison. The FE models follow the same DD structure as described in Equation (1); however, in this case, the variable values are substituted by the values minus their average in time, except in the case of binary variables.

Additionally, binary variables of years, states, and crossed effects of years and states were included in the models in order to control for federal and state policies over time, accounting for other shocks or local policies, such as the national program for the fortification of maize and wheat flour (established from 2014 onwards), the Bolsa Familia (implemented from 2003 onwards), and others. The control variables were maintained in the models, independently of statistical significance, so as to provide their effects throughout the results. Statistical analysis was performed using Stata software (Stata Corp., College Station, TX, USA, version 15.0).

## 3. Results

Trends in hospitalization rates and lengths of stay due to iron-deficiency anemia among children up to 4 years old were increasing during the period from 1998 to 2004, declining from 2005 to 2013, and started to rise again after 2014. Mortality rates, on the other hand, showed an irregular trend throughout the period from 1998 to 2019 (Table 2).

The initial adherence of municipalities to the PNSF (from 2005 onwards) showed an increasing pattern until 2011, followed by irregular trends in population coverage (especially considering the period post-decentralization after 2013), similarly to the adherence of municipalities to the NutriSUS (from 2014 onwards).

Hospitalization rates and lengths of stay due to infectious intestinal diseases among children up to 4 years old showed steady trends during the period from 1998 to 2006, then decreased to a lower level and maintained steady trends until 2019.

The application of intravenous iron per 1000 inhabitants showed an increasing trend for the majority of the period analyzed, whereas medical appointments at home (proxies for Family Health Strategy coverage) increased from 1998 to 2015, followed by a decrease until 2019. Population counts increased throughout the period considered, whilst municipal GDP per capita grew from 1998 to 2014, showing a slow recovery after 2016.

The results of the DD–OLS models indicate that PNSF coverage presented statistically significant effects on iron-deficiency anemia-related hospitalizations, decreasing the occurrence of hospitalization rates among children up to 4 years old, while no effect of its decentralization was identified.

Municipalities with higher GDP per capita and higher rates of intravenous iron application among preschool children had lower hospitalization rates, whilst larger municipalities had higher hospitalization rates due to iron-deficiency anemia among children up to 4 years old (Table 3).

Regarding NutriSUS coverage, the DD–OLS estimates showed a statistically significant improvement in children’s lengths of stay due to iron-deficiency anemia. The national level of length of children staying in hospitals, however, increased as a whole in the country as of 2014, but hospitalization and mortality rates remained the same. Population size showed a positive effect on length of stay in anemia-related hospitalizations of children.

Neither the PNSF nor NutriSUS coverage presented statistically significant effects on mortality rates attributable to iron-deficiency anemia, like the other variables tested. Hospitalization rates and lengths of stay due to infectious intestinal diseases among children did not present an influence on anemia-related health outcomes, and therefore were excluded from the models.

However, since the FE models are more robust, we consider as the final results the estimates available on Table 4. These results are basically the same as those on Table 3, but with greater coefficients in size, which suggests potential bias in the DD–OLS estimates.

The main changes occurred in the statistical significance of the crossed effect of PNSF decentralization and coverage, and of the effect of the intravenous iron application rate on children’s lengths of stay, showing that PNSF decentralization presented beneficial effects on municipalities with higher PNSF coverage, and that intravenous iron applications may be used supplementarily to the NutriSUS in the recovery of children with iron-deficiency anemia (Table 4).

In addition, FE estimates showed that, when comparing similar municipalities, intravenous iron application lost statistical significance to hospitalization rates, although it may have still had very small effects on mortality rates due to iron-deficiency anemia among children.

Finally, we analyzed municipalities’ heterogeneity based on the Brazilian Ministry of Health’s categorization of municipalities in extreme poverty. The results of the DD–FE estimates showed that the effects of the PNSF and the NutriSUS on hospitalization rates and lengths of stay due to iron-deficiency anemia remained statistically significant in non-poor municipalities, reducing hospitalizations and inpatient days, respectively. Intravenous iron applications also contributed to decreases in lengths of stay and mortality rates in non-poor municipalities.

On the other hand, municipalities in extreme poverty benefited exclusively from the NutriSUS to reduce lengths of stay and mortality due to iron-deficiency anemia in children up to 4 years old (Table 5).

## 4. Discussion

The results of the study show that severe health outcomes attributable to iron-deficiency anemia among children up to 4 years old still remained important public health concerns in Brazilian municipalities during recent decades. Although hospitalization rates and lengths of stay declined through the period from 2005 to 2013, they began to rise after 2014, whilst mortality rates maintained an irregular trend without significant decreases throughout the period from 1998 to 2019.

There is a lack of evidence in the literature specifically regarding hospitalizations of infants due to iron-deficiency anemia; the majority of studies referring to hospitalizations and iron-deficiency anemia refer to hospital-acquired anemia [23]. However, a recent study using administrative data from 52 children’s hospitals in the United States from 2004 to 2018 showed increasing admission rates attributable to iron-deficiency anemia in recent years (5 p.p. growth over the period 2016–2018, in comparison to the period 2004–2006) [24], similarly to the findings in our study.

In addition, a systematic analysis of the global burden of anemia indicated that children under 5 years old had the highest anemia prevalence worldwide, particularly in low- and middle-income regions, associated with increasing trends throughout the period from 1990 to 2010 [7].

There are substantial effects of iron supplementation in averting growth and developmental problems due to iron-deficiency anemia during early childhood [25], in addition to other short- and long-term health outcomes [10]. Our results indicated that Brazilian federal programs targeting iron-deficiency anemia among children up to 4 years old presented effects on different dimensions of the health problem: the PNSF reduced hospitalization rates, whilst the NutriSUS decreased the lengths of hospital stays attributable to anemia in Brazilian municipalities.

The fixed effect models showed that extreme measures to tackle iron-deficiency anemia at the hospital level—i.e., intravenous iron applications—were effective in reducing lengths of stay and mortality rates in Brazilian municipalities. Intravenous administration of iron is usually adopted for the treatment of adults with iron-deficiency anemia, with its utilization in children being unusual [26,27].

Furthermore, the effects of the micronutrient distribution programs were statistically significant in reducing hospitalizations and lengths of stay in non-poor municipalities, while Brazilian municipalities in extreme poverty only benefited from the adoption of the NutriSUS to reduce lengths of stay and mortality rates among children up to 4 years old.

Previous studies on the PNSF identified in the literature were conducted on small samples of children from two medium-sized municipalities in the southeastern region of Brazil in 2007, showing that the program increased the hemoglobin levels of children under two years old supplemented during a six-month period [28,29].

Regarding the NutriSUS, a pragmatic controlled trial with a small sample of children from 6 to 8 months old was conducted in one municipality in the northern region of Brazil, indicating that there was a statistically significant impact on plasma ferritin concentration after two months of multi-micronutrient supplementation [30].

On the other hand, a qualitative study conducted in one municipality in the southeastern region identified failures in the planning and execution of the NutriSUS, particularly due to low acceptance of the multi-micronutrient supplement by the children [31].

It is important to highlight that the previous evidence on the PNSF and NutriSUS emphasizes that the results showed negligible to null effects of the supplementation on the occurrence of iron-deficiency anemia in the sample of children participating in the studies [28,29,30], contrary to the findings in our study.

The differences in results obtained in the present study in comparison to evidence from previous studies may be attributable to several reasons: First, our study relies on nationwide information on the hospitalizations, inpatient days, and mortality of children up to 4 years old, representative at the population level, whilst other studies were performed on small samples of children in single municipalities from two Brazilian regions [28,29,30].

Second, our study gathered information throughout the period from 1998 to 2019, encompassing temporal trends in health outcomes attributable to iron-deficiency anemia before and after the implementation of supplementation through the PNSF and NutriSUS, whereas other studies monitored the supplementation of children during a limited timeframe of two to six months [28,29,30].

Finally, the main limitation of our study refers to the adoption of panel data of municipal-level information on supplementation and health outcomes due to iron-deficiency anemia, comprising an ecological study. On the other hand, it is important to emphasize the strength of the study, referring to the selection of modeling strategies based on difference-in-differences and fixed effects estimation procedures, which aimed at identifying the contributions of the programs towards children’s health at the population level, including diverse control variables to account for potential differences in local policies. The robustness of the models estimated allowed us to verify the complementarity of federal government programs designed to tackle iron-deficiency anemia in the country in recent decades, showing evidence of the need to invest in multiple strategies to address the problem in developing countries worldwide.

Most of the studies focusing on the subject are limited to the analysis of small-scale interventions [18,19], and there was a lack of evidence on the effects of programs to combat iron-deficiency anemia at the population level, especially referring to coverage of target population groups in smaller geographical areas [32]. Therefore, the present study aimed to fill the gap in the literature with data from two national programs implemented in Brazilian municipalities throughout recent decades.

## 5. Conclusions

Our results highlight the effect of the PNSF on the decrease in hospitalizations, on the one hand, and the effect of the NutriSUS on the reduction of lengths of stay on the other, indicating complementarity between the programs in tackling severe iron-deficiency anemia among children up to 4 years old in Brazilian municipalities. In addition, municipalities in extreme poverty benefited from the NutriSUS in terms of reduction of lengths of hospital stays and the mortality rates of children.

Nevertheless, in view of the shortcomings of the PNSF and NutriSUS in the reduction of mortality rates in non-poor municipalities, the operationalization of the PNSF and NutriSUS should be targeted for improvements throughout Brazilian municipalities, in order to achieve better results in the reduction of severe iron-deficiency anemia requiring hospitalization among children up to 4 years old.

## Figures and Tables

**Table 1:** Description of the variables of the study.

Dependent Variables						
Variable	Obs.	Mean	SD	Min	Max	Source
Hospitalization rate due to anemia among children ≤ 4 years old per 1000 individuals	122,257	0.0925	0.4662	0	25.64	DATASUS
Length of stay due to anemia among children ≤ 4 years old per hospitalization	122,257	0.7003	4.9824	0	1099	DATASUS
Mortality rate due to anemia among children ≤ 4 years old per 1000 individuals	122,257	0.0012	0.0593	0	12.66	DATASUS
Interest Variables						
PNSF relative coverage	122,257	0.0036	0.0256	0	1	MoH
NutriSUS relative coverage	122,257	0.0003	0.0083	0	1	MoH
Control Variables						
Intravenous iron application rate per 1000 inhabitants	122,257	0.5829	5.7804	0	266.90	DATASUS
Medical appointments at home coverage per 1000 inhabitants	122,257	101.9189	1675.94	0	299,249	DATASUS
Hospitalization rate due to infectious intestinal diseases among children ≤ 4 years old per 1000 individuals	122,255	14.1264	18.3011	0	938.56	DATASUS
Length of hospital stay due to infectious intestinal diseases among children ≤ 4 years old per 1000 individuals	122,257	3.0371	2.7256	0	374.00	DATASUS
Dummy of post year 2014 (1 if year ≥ 2014 and 0 c.c.)	122,257	0.2734	0.4457	0	1	MoH
Logarithm of GDP per capita	122,246	9.5349	0.7524	6.039	13.95	IBGE
Logarithm of population	122,257	9.4075	1.1480	6.567	16.32	DATASUS
Year dummies (20 variables)	122,257	-	-	0	1	IBGE
State dummies (26 variables)	122,257	-	-	0	1	IBGE
Year and state crossed dummies (520 variables)	122,257	-	-	0	1	IBGE

Obs. = number of observations; SD = standard deviation; Min = minimum value; Max = maximum value; PNSF = National Iron Supplementation Program; NutriSUS = Strategy of Food Fortification with Micronutrients for Children; GDP = gross domestic product; DATASUS = Department of Informatics from the Brazilian Unified Health System; MoH = Brazilian Ministry of Health; IBGE = Brazilian Institute for Geography and Statistics.

**Table 2:** Characteristics of the population (mean and standard errors), Brazil, 1998–2019.

Year	Health Outcomes of Anemia among Children			Variables of Interest		Control for Other Interventions		Control for Infectious Intestinal Diseases among Children		Other Control Variables	
	Hospitalization Rate	Length of Stay	Mortality Rate	PNSF Coverage	NutriSUS Coverage	Intravenous Iron Application Rate	Medical Appointments at Home Coverage	Hospitalization Rate	Length of Stay	GDP per Capita ^a^	Population ^b^
1998	0.0980	0.5985	0.0019	0.0000	0.0000	0.0559	2.6464	19.7696	3.9477	3.0357	29.4653
	(0.0066)	(0.0292)	(0.0005)	(0.0000)	(0.0000)	(0.0208)	(0.2618)	(0.2838)	(0.0273)	(0.0435)	(2.4012)
1999	0.1040	0.6146	0.0010	0.0000	0.0000	0.1145	3.7296	20.3145	3.9272	3.0165	29.7708
	(0.0064)	(0.0321)	(0.0003)	(0.0000)	(0.0000)	(0.0272)	(0.3112)	(0.2927)	(0.0374)	(0.0432)	(2.4098)
2000	0.1092	0.6488	0.0012	0.0000	0.0000	0.2848	6.2342	19.6697	3.8920	3.0709	30.8467
	(0.0076)	(0.0333)	(0.0004)	(0.0000)	(0.0000)	(0.0531)	(0.6336)	(0.2839)	(0.0782)	(0.0475)	(2.5167)
2001	0.0941	0.5638	0.0016	0.0000	0.0000	0.3596	8.2724	20.3738	3.7366	3.2221	31.0046
	(0.0059)	(0.0283)	(0.0006)	(0.0000)	(0.0000)	(0.0576)	(0.7193)	(0.3364)	(0.0621)	(0.0477)	(2.5148)
2002	0.1452	0.7652	0.0018	0.0000	0.0000	0.3449	9.6604	19.7639	3.6998	3.3483	31.4088
	(0.0090)	(0.0711)	(0.0004)	(0.0000)	(0.0000)	(0.052)	(0.6666)	(0.2754)	(0.0497)	(0.0515)	(2.542)
2003	0.1462	0.9765	0.0018	0.0000	0.0000	0.4504	8.7129	19.6349	3.5972	3.7048	31.8114
	(0.0079)	(0.201)	(0.0005)	(0.0000)	(0.0000)	(0.06)	(0.5128)	(0.2602)	(0.0340)	(0.0577)	(2.5658)
2004	0.1518	0.7681	0.0016	0.0000	0.0000	0.5107	9.9045	18.1428	3.4553	3.7607	32.6563
	(0.0075)	(0.0303)	(0.001)	(0.0000)	(0.0000)	(0.0743)	(0.5743)	(0.2511)	(0.0269)	(0.0618)	(2.6155)
2005	0.1309	0.7109	0.0016	0.0008	0.0000	0.5915	14.5686	17.6961	3.2997	3.7110	33.1029
	(0.0069)	(0.0303)	(0.0004)	(0.0003)	(0.0000)	(0.087)	(0.7646)	(0.2491)	(0.0267)	(0.0615)	(2.6416)
2006	0.1260	0.6861	0.0007	0.0023	0.0000	0.5205	20.5411	18.4411	3.2618	3.9290	33.5677
	(0.0072)	(0.028)	(0.0002)	(0.0003)	(0.0000)	(0.0737)	(0.798)	(0.2645)	(0.0242)	(0.0627)	(2.6692)
2007	0.1226	0.7205	0.0006	0.0082	0.0000	0.0006	27.0527	13.1613	3.0689	4.2933	33.0675
	(0.0059)	(0.0304)	(0.0002)	(0.0005)	(0.0000)	(0.0004)	(1.0626)	(0.2012)	(0.0272)	(0.0677)	(2.6516)
2008	0.1094	1.4292	0.0010	0.0048	0.0000	0.6223	187.2172	14.5249	2.9592	4.5553	34.0771
	(0.0063)	(0.1155)	(0.0007)	(0.0003)	(0.0000)	(0.0722)	(39.6289)	(0.2246)	(0.0234)	(0.0718)	(2.6874)
2009	0.0840	0.9826	0.0005	0.0058	0.0000	0.6832	185.9675	12.8013	2.8555	4.5975	34.4080
	(0.0053)	(0.0786)	(0.0002)	(0.0003)	(0.0000)	(0.0796)	(15.2829)	(0.2105)	(0.0263)	(0.0662)	(2.7052)
2010	0.0686	0.7962	0.0003	0.0139	0.0000	0.7187	194.4540	15.2963	2.7635	5.0431	34.2778
	(0.0047)	(0.0687)	(0.0002)	(0.0006)	(0.0000)	(0.0843)	(18.8597)	(0.2531)	(0.0241)	(0.079)	(2.7227)
2011	0.0624	0.6664	0.0003	0.0120	0.0000	0.7455	182.8117	10.567	2.5321	5.4235	34.5695
	(0.0048)	(0.0522)	(0.0001)	(0.0006)	(0.0000)	(0.0875)	(17.1061)	(0.2006)	(0.024)	(0.0897)	(2.7409)
2012	0.0603	0.6238	0.0001	0.0097	0.0000	0.8520	234.3024	11.1797	2.5926	5.6082	34.8435
	(0.0046)	(0.0548)	(0.0001)	(0.0005)	(0.0000)	(0.0979)	(20.6780)	(0.2055)	(0.0372)	(0.0944)	(2.7585)
2013	0.0509	0.4668	0.0002	0.0038	0.0000	0.8026	268.1921	9.7738	2.5147	5.8288	36.0921
	(0.0047)	(0.0447)	(0.0002)	(0.0003)	(0.0000)	(0.0938)	(57.1821)	(0.1946)	(0.0284)	(0.0902)	(2.8494)
2014	0.0586	0.5191	0.0022	0.0013	0.0009	0.8240	257.7224	10.5433	2.5036	5.8822	36.4037
	(0.0058)	(0.0433)	(0.0020)	(0.0003)	(0.0002)	(0.0926)	(31.8909)	(0.2136)	(0.0262)	(0.0903)	(2.8694)
2015	0.0601	0.5776	0.0002	0.0003	0.0007	0.6949	263.0202	8.1406	2.4727	5.5884	36.7056
	(0.0050)	(0.0458)	(0.0002)	(0.0002)	(0.0002)	(0.078)	(52.9045)	(0.1744)	(0.0285)	(0.0765)	(2.8888)
2016	0.0664	0.5951	0.0004	0.0002	0.0000	0.8809	126.3717	9.2835	2.5278	5.6216	36.9985
	(0.0069)	(0.0443)	(0.0003)	(0.0002)	(0.000)	(0.1031)	(13.9784)	(0.2151)	(0.0309)	(0.0709)	(2.9078)
2017	0.0615	0.5450	0.0007	0.0019	0.0006	0.8292	112.9265	7.4713	2.4121	5.7199	37.2820
	(0.0052)	(0.0476)	(0.0005)	(0.0003)	(0.0002)	(0.0949)	(13.2853)	(0.1775)	(0.0309)	(0.073)	(2.9263)
2018	0.0596	0.5813	0.0002	0.0125	0.0027	0.8632	49.8198	7.3632	2.3791	5.6633	37.4318
	(0.0053)	(0.0482)	(0.0001)	(0.0007)	(0.0003)	(0.0953)	(7.9060)	(0.1903)	(0.0289)	(0.0719)	(2.9474)
2019	0.0652	0.5696	0.0069	0.0019	0.0012	1.0577	64.1115	7.1266	2.4529	5.8586	37.7284
	(0.0056)	(0.0520)	(0.0025)	(0.0002)	(0.0002)	(0.1067)	(25.1662)	(0.1710)	(0.0313)	(0.0741)	(2.9673)

Obs.: ^a^ GDP in 1000 US dollars; ^b^ Population count in 1000 inhabitants.

**Table 3:** Coefficients of the DD–OLS estimates on the effects of the PNSF and NutriSUS on anemia-related outcomes among children, Brazil, 1998–2019.

Variables	Anemia-Related Outcomes among Children ≤ 4 Years Old		
	Hospitalization Rate	Length of Stay	Mortality Rate
PNSF relative coverage	−0.0895 **	0.8655	0.0111
	(0.037)	(0.873)	(0.012)
NUTRISUS relative coverage	0.0538	−0.6216 *	−0.0035
	(0.107)	(0.376)	(0.003)
Dummy of post year 2014 (1 if year ≥ 2014, and 0 c.c.)	0.0097	1.4685 *	−0.0042
	(0.113)	(0.882)	(0.004)
Crossed effect of PNSF decentralization and coverage	0.0926	−1.2336	−0.0136
	(0.057)	(0.907)	(0.012)
Population (log)	0.0069 ***	0.2820 ***	−0.0003
	(0.002)	(0.025)	(0.000)
GDP per capita (log)	−0.0097 ***	−0.0095	0.0002
	(0.004)	(0.054)	(0.001)
Intravenous iron application rate	−0.0004 **	−0.0038	−0.0000
	(0.000)	(0.002)	(0.000)
Medical appointments at home coverage	−0.0000	0.0000	0.0000
	(0.000)	(0.000)	(0.000)
Observations	122,246	122,246	122,246
R^2^	0.032	0.041	0.003

Obs.: PNSF = National Iron Supplementation Program; NutriSUS = Strategy of Food Fortification with Micronutrients for Children; GDP = gross domestic product. Robust standard errors are in parentheses, clustered by municipality. *** *p* < 0.01, ** *p* < 0.05, * *p* < 0.1. Models include controls for states, years, and the crossed effects of states and years.

**Table 4:** Coefficients of the DD–FE estimates on the effects of the PNSF and NutriSUS on anemia-related outcomes among children, Brazil, 1998–2019.

Variables	Anemia-Related Outcomes among Children ≤ 4 Years		
	Hospitalization Rate	Length of Stay	Mortality Rate
PNSF relative coverage	−0.1028 **	1.1679	0.0138
	(0.049)	(0.730)	(0.013)
NUTRISUS relative coverage	0.0535	−0.9483 **	−0.0033
	(0.089)	(0.401)	(0.003)
Dummy of post year 2014 (1 if year ≥ 2014, and 0 c.c.)	0.0180	1.6426 *	−0.0044
	(0.113)	(0.889)	(0.004)
Crossed effect of PNSF decentralization and coverage	0.0406	−1.6445 **	−0.0172
	(0.069)	(0.796)	(0.013)
Population (log)	−0.0214	−0.4371 *	0.0005
	(0.020)	(0.224)	(0.001)
GDP per capita (log)	−0.0151 *	−0.0682	0.0004
	(0.008)	(0.110)	(0.001)
Intravenous iron application rate	−0.0001	−0.0168 ***	−0.0000 **
	(0.000)	(0.005)	(0.000)
Medical appointments at home coverage	0.0000	0.0000	0.0000
	(0.000)	(0.000)	(0.000)
Observations	122,246	122,246	122,246
R^2^	0.020	0.030	0.003
Number of municipalities	5570	5570	5570

Obs.: PNSF = National Iron Supplementation Program; NutriSUS = Strategy of Food Fortification with Micronutrients for Children; GDP = gross domestic product. Robust standard errors are in parentheses, clustered by municipality. *** *p* < 0.01, ** *p* < 0.05, * *p* < 0.1. Models include controls for states, years, and the crossed effects of states and years.

**Table 5:** Coefficients of the DD–FE estimates on the effects of the PNSF and NutriSUS on anemia-related outcomes among children, according to poverty categorization, Brazil, 1998–2019.

Variables	Anemia-Related Outcomes among Children ≤ 4 Years					
	Extreme Poverty Municipalities			Other Municipalities		
	HR	LS	MR	HR	LS	MR
PNSF relative coverage	−0.07953	1.73743	0.03729	−0.11776 **	0.92390	0.00112
	(0.098)	(1.510)	(0.036)	(0.054)	(0.767)	(0.002)
NutriSUS relative coverage	0.31444	−1.51201 *	−0.00857 *	−0.03409	−0.65482 *	−0.00102
	(0.201)	(0.900)	(0.005)	(0.049)	(0.354)	(0.002)
Dummy of post year 2014 (1 if year ≥ 2014, and 0 c.c.)	−0.20989	1.17987	−0.01433	0.10127	1.84768	−0.00065
	(0.360)	(0.749)	(0.015)	(0.076)	(1.183)	(0.001)
Crossed effect of PNSF decentralization and coverage	−0.02158	−2.38914	−0.04663	0.08478	−1.35670 *	−0.00094
	(0.116)	(1.664)	(0.037)	(0.087)	(0.818)	(0.002)
Population (log)	−0.05860	−0.69238 ***	−0.00072	−0.00245	−0.39303	0.00052
	(0.049)	(0.209)	(0.002)	(0.020)	(0.270)	(0.001)
GDP per capita (log)	−0.00425	−0.24493 *	−0.00131	−0.01828 *	−0.02805	0.00108
	(0.014)	(0.129)	(0.001)	(0.010)	(0.141)	(0.001)
Intravenous iron application rate	−0.00010	−0.00891	−0.00004	−0.00014	−0.01718 ***	−0.00003 **
	(0.001)	(0.007)	(0.000)	(0.000)	(0.006)	(0.000)
Medical appointments at home coverage	0.00000	0.00001 **	−0.00000	0.00000	−0.00000	0.00000
	(0.000)	(0.000)	(0.000)	(0.000)	(0.000)	(0.000)
Observations	34,774	34,774	34,774	87,472	87,472	87,472
R^2^	0.044	0.039	0.016	0.017	0.060	0.003
Number of municipalities	1582	1582	1582	3988	3988	3988

Obs.: HR = hospitalization rate; LS = length of stay; MR = mortality rate; PNSF = National Iron Supplementation Program; NutriSUS = Strategy of Food Fortification with Micronutrients for Children; GDP = gross domestic product. Robust standard errors are in parentheses, clustered by municipality. *** *p* < 0.01, ** *p* < 0.05, * *p* < 0.1. Models include controls for states, years, and the crossed effects of states and years.

## Data Availability

Data supporting reported results were gathered in publicly available datasets from the platform of the Brazilian Ministry of Health (MoH: https://sisaps.saude.gov.br/micronutrientes/, last access on: 15 March 2021), the Department of Informatics of the Brazilian Unified Health System (DATASUS: http://www2.datasus.gov.br/DATASUS/index.php?area=0202&id=11633, last access on: 15 March 2021), and the Brazilian Institute for Geography and Statistics (IBGE: https://www.ibge.gov.br/estatisticas/economicas/contas-nacionais/9088-produto-interno-bruto-dos-municipios.html?t=series-historicas, last access on: 15 March 2021).
